# Effects of Substrate Composition on the Growth Traits of Grafted Seedling in Macadamia (*Macadamia integrifolia*) Nuts

**DOI:** 10.3390/plants13121700

**Published:** 2024-06-19

**Authors:** Qiujin Tan, Chunheng Zhou, Peng Xu, Xiyun Huang, Zhenzhen Pan, Yuanrong Wei, Wenlin Wang, Lifeng Wang

**Affiliations:** 1 Guangxi South Subtropical Agricultural Research Institute, Longzhou 532415, Chinahuangxiyuan04@gxaas.net (X.H.);; 2 Rubber Research Institute, Chinese Academy of Tropical Agricultural Sciences, Haikou 571101, China; 3 Guangxi Academy of Agricultural Sciences, Nanning 530007, China

**Keywords:** grafted seedling, growth, *Macadamia integrifolia*, substrate, surviving rate

## Abstract

Macadamia nut plantings in China are expanding year by year. In order to breed and promote superior varieties, this study analyzed the effects of different rootstocks and scions on the survival rate of grafted seedlings, and then selected the best substrate composition for plant growth. The results showed that the survival rate of the HAES788 variety as rootstock and Guire No. 1 as scion was the highest, reaching 96%. The optimal grafting time in December was better than that in March. Furthermore, among 16 substrate formulations, T12, T13, T15, and T16 had advantages of agglomerated soil and more well-developed root systems compared to the CK made of loess. The plant height, stem diameter, leaf length, leaf width, and dry weight of the aboveground and underground parts of the grafted seedlings planted in these substrate formulations were significantly higher than those plants planted in the CK. In addition, the substrate formulations T12, T13, T15, and T16 significantly improved the organic matter, total nitrogen, and total potassium content of the substrate soils, but little improvement was observed for total phosphorus content after 13 months. Overall, macadamia grafting times are best in December, with HAES788 and Guire No. 1 being the best rootstock and scion. The optimal substrate formulations are T12, T13, T15, and T16. This study provides a solid foundation for the production of high-quality macadamia plants.

## 1. Introduction

Grafting is a widely used practice for the asexual propagation of fruit trees [1]. It can increase production, physico-chemical characteristics, nutritional quality, and the yield of fruit [2,3]. The selection of rootstock and scion is the top priority in fruit tree grafting because of the differential responses between rootstock and scion in this process [4]. This is because rootstock mediates transcriptional regulation and the accumulation of secondary metabolites [5,6]. Furthermore, grafting also affects endogenous hormone content [7] and oxidative defense reactions [8], and can even enhance resistance to biotic and abiotic stresses [9,10]. In the grafting technique, the substrate or matrix composition is important because it affects the hormonal crosstalk, protein and small-molecule movement, nutrient uptake, and transport in the grafted trees [1]. Meanwhile, it has also been found that Breadfruit (*Artocarpus altilis*) scions growing on Lakoocha (*Artocarpus lakoocha*) rootstocks enhance total flavonoid contents and the expression of flavonoid biosynthetic genes, such as *AaCHS* and *AaDFR* [11]. Cabernet Sauvignon grapes grafted on different rootstocks and rootstock combinations enhance saline–alkaline stress resistance capacity [12]. This technique can be used in transgenic donor rootstocks and applied to a wide range of breeding programs of fruit and crop [13]. It has found rootstock-mediated carbohydrate metabolism, nutrient contents, and physiological modifications in regular and alternate mango scion varieties [14].

Macadamia nut, also known as *Macadamia integrifolia* Maiden and Betche, is an evergreen fruit tree of the family Proteaceae, native to Australia [15]. Macadamia nut kernels are rich in oil nutrition [16], can be used as medicine and food, and have the reputation of being “the king of dried fruits” [17]. Macadamia species are generally grown in an environment with an average annual temperature ranging from 60 °F to 85 °F, an annual precipitation of more than 40 and 60 inches per year, spread evenly, an altitude of less than 1300 m, a soil depth of more than 1m, and a soil pH value ranging from 4.5 to 6.5 [18]. Macadamia nuts begin to bear fruit after 3~4 years after planting and enter the bumper yield period in 8~10 years, and the economic benefits increase year on year [19]. Macadamia cultivation is a research hotspot; high yield is achieved by cultivating dwarf rootstock varieties, combined with pruning and dwarfing trees. Macadamia seedlings mainly include cuttings, grafts, and high-altitude strips, but their wood is brittle and hard, and the survival rate from grafting is low. The grafting method is used for macadamia seedlings at home and abroad, and the techniques used differ under different ecological and geographical conditions. After years of practice, macadamia nuts are now commonly grafted using splitting, tongue, ventral, and dorsal grafting. Our previous studies involved Guire No. 1 macadamia nuts grafted via branch-belly grafting, and the grafting survival rate was the highest (86.40%), followed by cutting grafting, with a grafting survival rate of 70.60%. It was found that the grafting period, grafting method, rootstock quality, and rootstock combination are the main factors affecting the survival rate of grafted seedlings. The survival rate differs according to the rootstock combinations as the adaptability, stress resistance, and high yield performance will be different [19,20]. Moreover, the nutritional contents of root soil have a significant effect on seedling growth. Another important factor is the seedling substrate [21,22]. The substrate composition affects the phenotype, survival rate, and biomass of grafted seedlings. For instance, auxin (IAA), cytokinin (CK), abscisic acid (ABA), gibberellic acids (GAs), and brassinosteroids (BRs) have been shown to have an impact on the scion vigor of rootstocks [23]. Many fruit wastes can be used as a substrate, RHA:peat:vermiculite:perlite = 4:4:1:1 (volume ratio) significantly enhanced substrate ventilation and positively influenced the stem diameter of cucumber and melon plants [24]. A significant correlation between soil enzyme activities and physical and chemical indicators has been established. Actual and potential dehydrogenase, acid phosphatase, and catalase activities were found to be related to tillage and crop rotation [25].

In summary, the purpose of this study was, firstly, to screen for macadamia varieties with strong grafting affinity for rootstock and scion, and to explore the optimal rootstock combination. Secondly, we investigated the growth of grafted seedlings through 16 different substrate compositions and compared their effects on the biomass of seedlings such as plant height, stem diameter, leaf length, and leaf width, and then selected the most favorable substrate ratio for seedling growth. These results will provide useful guidance for macadamia plant production.

## 2. Results

### 2.1. Effect of Macadamia Scion and Rootstock on Survival Rate of Grafted Plants

**Figure 1:**
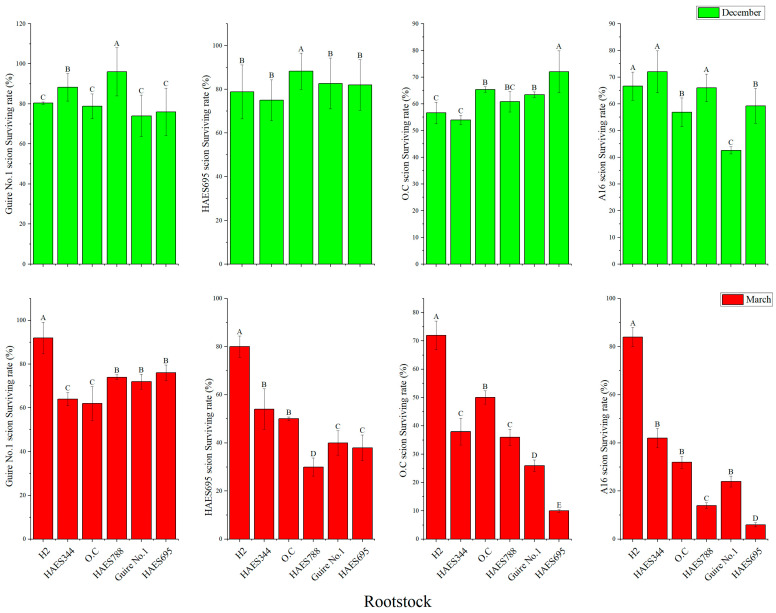
Effects of different scion and rootstock groups on the survival rate of grafted plants. Note, different uppercase letter means significant level (*p* < 0.01).

The main macadamia varieties H2, HAES344, O.C, HAES788, Guire No. 1, and HAES695 were used as rootstocks, and Guire No. 1, HAES695, O.C, and A16 were used as scions. Bud grafting experiments were carried out in December and March of the following year, respectively. The results showed that the average grafting survival rates of Guire No. 1, HAES695, O.C, and A16 scions in December were 82.25%, 73.33%, 77.13%, and 48.67%, respectively. The average grafting survival rates of Guire No. 1, HAES695, O.C, and A16 scions in March were 62.04%, 38.67%, 60.56%, and 33.67%, respectively. The survival rate was ranked as Guire No. 1 > HAES695 > O.C > A16. The survival rate in December was significantly higher than that in March. The best combinations were HAES788 as rootstock and Guire No. 1 as scion in December, with a survival rate of 96% (Figure 1).

### 2.2. Effect of Different Substrate Composition on the Growth of Grafted Plants

#### 2.2.1. Root Phenotypes of Grafted Plants Cultivated with Different Substrate Compositions

**Figure 2:**
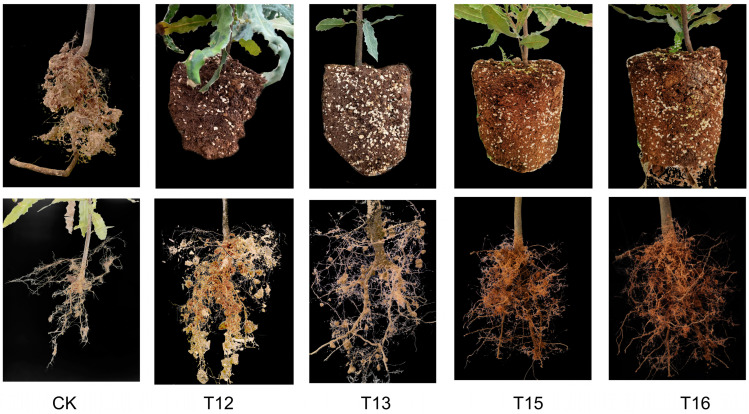
Root phenotypes of grafted plants planted in CK, T12, T13, T15, and T16 substrate compositions.

In order to reveal the effect of 16 substrate compositions (Table 1) on the growth and development of the grafted plants, the growth changes in the plants HAES788 as rootstock and Guire No. 1 as scion were continuously observed once every three months to determine the best substrate composition. As can be seen from Figure 2, the root growth of the grafted plants planted in substrate compositions T12, T13, T15, and T16 was significantly higher than that of the seedlings planted in CK, which was made of loess. These four substrate compositions were the best formulas. The main difference was that the root system had less soil attachment and there was less of a root system of the shoots in the CK substrate. The root soil of the T12-, T13-, T15-, and T16-treated seedlings was tightly fixed, the taproot of the grafted seedlings was thick, and the number of fine roots increased significantly (Figure 2).

#### 2.2.2. Effect of Substrate Composition on the Growth of Graft Plants

**Figure 3:**
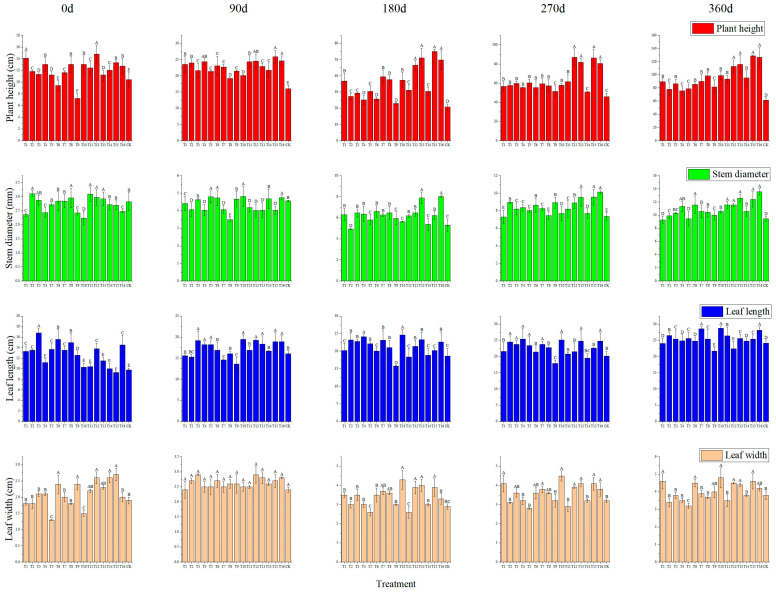
Effects of 16 substrate compositions on the plant height, stem diameter, leaf length, and leaf width of grafted plants. The colors of the heat map squares represent the results of data normalization. Red and Green mean high and low values, respectively. Note, different uppercase letter means significant level (*p* < 0.01).

As can be seen from Figure 3, the plant height continued to increase with the extension of the cultivation time, and the plant length of the plants planted in CK increased from 10.4 cm to 61.41 cm. The grafted seedlings cultivated in T12, T13, T15, and T16 substrate compositions increased from 14.8, 11.2, 13.3, and 12.7 cm to 112.56, 115.85, 128.48, and 126.58 cm, respectively. The stem diameter of the plants planted in CK grew from 2.82 cm to 9.45 cm. The stem diameter of the grafted seedlings cultivated in T12, T13, T15, and T16 substrate compositions increased from 2.98, 2.93, 2.48, and 2.82 cm to 11.56, 12.58, 12.41, and 13.57 cm, respectively. The leaf length of the plants planted in CK ranged from 9.8 cm to 24.13 cm. The plants planted in the T12, T13, T15, and T16 substrate compositions increased from 13.8, 11.5, 9.3, and 14.5 cm to 22.4, 25.63, 25.43, and 28.14 cm, respectively. The leaf width of the plants planted in CK increased from 1.9 cm to 3.8 cm. The leaf width of the plants planted in the T12, T13, T15, and T16 substrate compositions increased significantly from 2.6, 2.3, 2.7, and 2.00 cm to 4.5, 4.4, 4.6, and 4.2 cm, respectively (*p* < 0.05).

**Figure 4:**
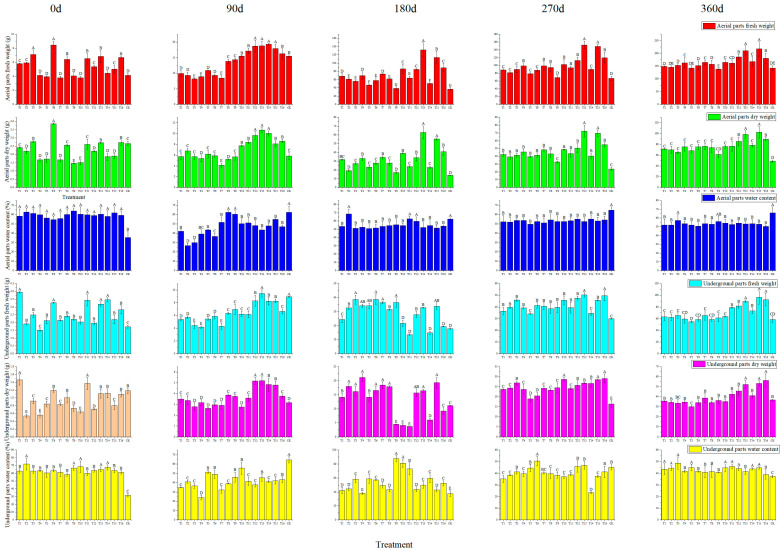
Effects of substrate composition on the aerial parts’ fresh weight, aerial parts’ dry weight, aerial parts’ water content, underground parts’ fresh weight, underground parts’ dry weight, and underground parts’ water content of grafted plants. Note, different uppercase letter means significant level (*p* < 0.01).

As can be seen from Figure 4, with the extension of the cultivation time, aerial parts’ fresh weight, aerial parts’ dry weight, underground parts’ fresh weight, and underground parts’ dry weight of the plant continued to increase. The aerial parts’ fresh weight of the plants planted in CK increased from 4.13 g to 142.36 g. The plants planted in the T12, T13, T15, and T16 substrate compositions increased from 5.37, 6.83, 5.01, and 6.68 g to 186.36, 210.32, 217.53, and 180.36 g, respectively, with a significant difference (*p* < 0.01). The aerial parts’ dry weight of the plants planted in CK increased from 2.67 g to 48.62 g. The plants planted in the T12, T13, T15, and T16 substrate compositions increased from 2.21, 2.71, 1.87, and 2.73 g to 85.63, 98.47, 102.36, and 89.47 g, respectively, with a significant difference (*p* < 0.01). The aerial parts’ water content of the plants planted in CK increased from 35.35% to 65.85%. The plants planted in the T12, T13, T15, and T16 substrate compositions decreased from 58.85, 60.32, 61.87, and 59.13% to 54.05, 53.18, 52.94, and 50.39%, respectively, with a significant difference (*p* < 0.01).

The underground parts’ fresh weight of the plants planted in CK increased from 1.43 g to 58.64 g. The plants planted in the T12, T13, T15, and T16 substrate compositions increased from 1.96, 3.19, 2.2, and 2.82 g to 81.62, 89.64, 96.35, and 92.45 g, respectively, with a significant difference (*p* < 0.01). The underground parts’ dry weight of the plants planted in CK increased from 1.18 g to 36.74 g. The plants planted in the T12, T13, T15, and T16 substrate compositions increased from 0.71, 1.11, 0.80, and 1.09 g to 45.63, 52.36, 53.24, and 56.32 g, respectively, with a significant difference (*p* < 0.01). The underground parts’ water content of the plants planted in CK increased from 31.79% to 37.64%, while that of the plants planted in the T12, T13, T15, and T16 substrate compositions decreased from 63.78, 65.20, 63.64, and 61.35% to 44.09, 41.58, 44.74, and 39.08%, respectively, with a significant difference (*p* < 0.01).

#### 2.2.3. Nutrient Composition Changes in Different Substrate Compositions after 13 Months Planting

**Figure 5:**
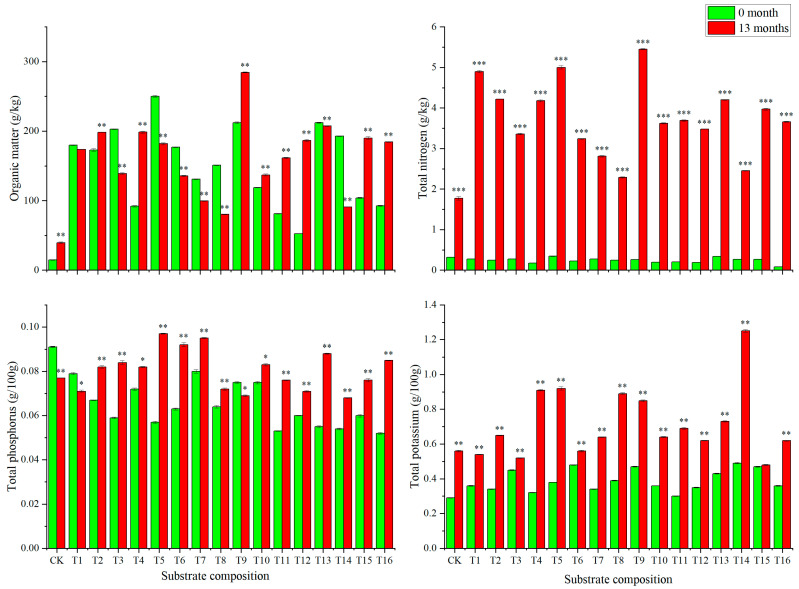
Effect of substrate compositions on nutrient composition after 13 months of planting with grafted plants. *, **, and *** mean significant difference between 0 months and 13 months at *p* < 0.05, *p* < 0.01, and *p* < 0.001 level, respectively.

In order to analyze the effective nutrient composition of the substrates, we compared and analyzed the main substrate nutrients after 13 months of planting grafted plants compared to that of 0 months. As can be seen from Figure 5, after 13 months, the organic matter, total nitrogen, total phosphorus, and total potassium contents in the CK changed from 14.6 g kg^−1^, 0.32%, 0.09%, and 0.29% to 39.51 g kg^−1^, 1.77%, 0.08%, and 0.56%, respectively. Substrate compositions T1–T16 had significantly higher levels of organic matter and total nitrogen than those in CK. The organic matter content was 2.03~7.21, the total nitrogen was 1.29~3.07 times that of the CK, the total phosphorus was 0.88~1.26 times that of the control, and the total potassium was 0.85~2.23 times that of the CK. Substrate composition T9 had the highest organic matter and total nitrogen contents, which were 284.59 g kg^−1^ and 5.45 g kg^−1^, respectively. The total phosphorus of substrate composition T5 was the highest, which was 0.097 g/100 g, and the total potassium of substrate composition T13 was the highest, which was 0.73 g/100 g.

## 3. Discussion

There are significant differences in the growth and development of the different varieties of macadamia nut, which have been proved by GWAS and other techniques [26,27]. For instance, compared with Guire No. 1, the plant height of H2 was higher, the diameter of the underground part of HAES695 was thicker, the taproot of A16, O.C, and O.V was longer, and the fresh weight of H2 was larger [28]. When determining differences in nut size and quality, it was found that small kernels had higher concentrations of many essential nutrients [29]. The HAES741 [30] genome analysis provides a good basis for yield component traits in macadamia [31]. Due to pollen limitation and xenia effects of macadamia nut [32], grafting is the commonly used method for macadamia nut plantation. Our previous studies showed that 12 months after grafting, the survival rate of grafting plants with different rootstock combinations was between 75.33% and 84.67%. A comprehensive comparison of scion growth, taproot length, fresh plant weight, and the seedling strength index of different rootstock combinations showed that H2, A16, and HAES695 exhibited good growth and development traits when they were rootstocks. There are general differences in the seedling growth and development rate of different macadamia nut varieties, and the survival rate of conventional grafting is about only 50.00%. In this study, we found HAES788 and Guire No. 1 to be the best rootstock and scion (Figure 1). The survival rate of grafted seedlings of Guire No. 1 as a scion with HAES788 as the rootstock could reach more than 96.00%. The number of leaves left in the grafted rootstock was positively correlated with the survival rate and physiological growth of the scion, and the greater the number of leaves of the rootstock, the higher the survival rate of the grafted plants. This grafting technology can improve the survival rate of macadamia seedlings, shorten the seedling rearing time, and reduce the seedling breeding cost. Grafting time, grafting site, and grafting technique all affect the grafted plants survival rate of macadamia nuts [29]. The grafting time of macadamia nuts is generally from late October to early February of the following year, and some studies have found that the survival of different varieties of rootstocks varies significantly when grafted at different times. The grafting time in this study was 12 months, which may have influenced the survival rate. In Kinnow mandarin, researchers observed that the scion attained the maximum plant height, and the number of fruits, total yield, and other traits on *jattikhatti* rootstock [33]. It has been shown that the complex interactions between scion and rootstock can regulate plant development and structure [34]. The scion genotype highly influences the rootstock microbiome in *Rosa* [35]. These results were also found in other tropical crops, such as rubber tree [36] and watermelon [5]. Previous studies identified the physiological and molecular mechanisms of rootstock control scion vigor, such as in apple, litchi, pear, and citrus [23]. These were related to endogenous hormones [7] and rhizosphere microbiome compositions [35]. These results were consistent with those of this study. Further research is needed to analyze the physiological traits of grafted macadamia nuts, such as photosynthetic rate, soluble sugar contents, etc. [31].

The cultivation of fruit trees can not only obtain fruit but also obtain wood waste, which can be used as a substrate for planting seedlings [37]. The ideal substrate for macadamia plants is deep, well-drained soil rich in organic matter with a slightly acidic to neutral pH. On the other hand, substrate composition is very important for the growth of grafted seedlings and the grower’s income [25]. For example, along with the cultivation, the particulate soil organic matter changes accordingly [38]. Scion and rootstock transportation modulates nitrate uptake capacity [26]. The biomass partitioning of macadamia with high manganese and low phosphorus concentrations showed that the dry weight of roots, stems, branches, and leaves accounted for 14–20%, 19–30%, 36–52%, and 12–18% of the total plant weight, respectively [35]. Combining economic and nutritional factors, in this study, we found that mixed substrate compositions enhanced the plant height, aerial parts’ fresh weight, and other characterizations of the grafted seedlings (Figure 3 and Figure 4). Among them, substrate compositions T12, T13, T15, and T16 were the best (Figure 2, Figure 3 and Figure 4). The reason for this is that these substrates significantly increase the soil structure and nutrient composition, such as organic matter, total nitrogen, and total potassium (Figure 5). It was also found that grafting plants also affects the rootstock rhizosphere microbiome assembly. Research on apple and pear’s rhizosphere microenvironment showed that rhizosphere microenvironmental parameters, ammonia nitrogen content, and soil pH were closely related to the soil microbial community. The soil microbial utilization of six C sources, nitrate nitrogen content, and invertase activity were negatively correlated with *Ambiguous* species and *Alternaria* [39]. Our results also showed that after 13 months, the substrate composition changed dramatically compared to that at 0 months. These may be related to microbiome feedback during macadamia plant growth in these substrates [40]. Tropical peat soils are generally defined as soils formed by the accumulation of partially decayed woody plant materials under waterlogged condition [41]. Perlite and wood bran are commonly used in fruit and vegetables growth substrates [7]. These were more easily obtained and lighter than loess and suitable for macadamia plant cultivation. Our results show that the substrate compositions were a four-volume ratio of peat soil, which provides enough organic content for grafted plant growth. In contrast, loess is a sedimentary deposit composed largely of silt-size grains that are loosely cemented by calcium carbonate. This shows a lack of organic matter and nutrients (Figure 2). In summary, plants planted in T12, T13, T15, and T16 substrate compositions had the advantages of agglomerated soil and more well-developed root systems compared to plants planted in loess.

## 4. Materials and Methods

### 4.1. Materials

The experiment was performed in the macadamia resource garden of the experimental station of the Guangxi Subtropical Crop Research Institute (106°79′85″ E, 22°34′13″ N). The 7 macadamia varieties HAES695, A16, HAES788, H2, Guire No. 1 (selected and bred at this station) [42], HAES344, and O.C were used as materials. The seeds were picked on 28 September 2021, and the rootstock seedlings were cultivated in non-woven bags with a size of 10.5 cm × 15 cm.

### 4.2. Seedling Cultivation

On the day of picking, the outer pericarp of the ripe fruit was removed and dried naturally for 2 days. Then, the seeds were soaked in 500~1000× Carbendazim (CAS number: 10605-21-7, Merck KGaA, Darmstadt, Germany) for 24 h, and after some of the shells were cracked, they were sown on a sand bed with a thickness of about 40 cm and a width of about 1 m on 1 October 2021.

### 4.3. Grafted Seedling Survival Rate Test

On 1 December 2022, the seedlings of H2, HAES344, O.C, HAES695, HAES788, and Guire No. 1 were used as rootstocks, and Guire No. 1, O.C, A16, and HAES695 were used as scions. Each rootstock combination was repeated 3 times. After one year of growth, the surviving rates were calculated to identify the best rootstock and scion groups.

### 4.4. Analysis and Identification of Best Substrate Composition

To investigate the growth of seedlings grown using 16 different substrate compositions (Table 1), and to compare their effects on the biomass of the seedlings of HAES788 and Guire No. 1 as rootstock and scion, parameters including plant height, stem diameter, leaf length, leaf width, aerial parts’ fresh weight, aerial parts’ water content, aerial parts’ dry weight, underground parts’ fresh weight, underground parts’ dry weight, and underground parts’ water content were measured. In order to select the best substrate compositions for seedling growth, five seedlings were randomly selected from each substrate with consistent growth and were labeled, with the plant height, stem diameter, leaf length, and leaf width of the seedlings measured every three months using a ruler and vernier calipers.

When measuring aerial parts’ fresh weight, aerial parts’ water content, and aerial parts’ dry weight, the stems and leaves were first cut and packed into a file bag, the fresh weight was weighed with an electronic balance MCM40K3 (Sartorius Lab Instruments GmbH & Co., KG, Goettingen Germany), and then the sample was put into a VT6130M oven (Thermo Fisher Scientific Inc., Shanghai, China) for 30 min at 105 °C. Following this, the sample was dried in a 75 °C constant-temperature drying oven for 24~32 h, and then the dry weight was measured. The roots were dug out and rinsed with water, put into an oven for 30 min at 105 °C, and then dried in a 75 °C constant-temperature drying oven for 24~32 h before the dry weight was measured.

### 4.5. Measurement of Substrate Nutrient Composition

The nutrient composition, organic matter, total nitrogen, total phosphorus, and total potassium contents of the different substrate compositions were measured. For organic matter measurements, 10 g subsamples of soil were dispersed in 30 mL of 5 g L^−1^ sodium hexametaphosphate (CAS No. 10124-56-8, Thermo Fisher Scientific Inc., Shanghai, China) via shaking for 15 h on an OHAUS™ Reciprocating Shaker (Thermo Fisher Scientific Inc., Shanghai, China). The dispersed soil samples were passed through a 53 µm sieve and, after rinsing several times with water, the material that was retained on the sieve was dried at 50 °C overnight. The soil slurry passing through the sieve contained the mineral-associated and water-soluble C and N. Water in the slurry was evaporated in a forced-air oven at 50 °C and the dried sample was ground with a mortar and pestle and analyzed for total organic C [38]. To measure total nitrogen, put 20 mL distilled water and 20 g soil into a 1000 mL distillation flask. Add 100 mL of potassium permanganate (0.32%) and 100 mL of sodium hydroxide solution (2.5%) to the flask. Stopper the flask immediately and start distillation. The tip of the condenser should dip into the 20 mL of boric acid solution in the beaker. On heating, ammonia will be released, which will be absorbed in the boric acid. The original wine’s red/pink red color turns to green with the absorption of ammonia. Collect nearly 100 L of the distillate in about 30 min and add to 1 L of 0.02 N H_2_SO_4_ to obtain the original pink red wine color and record the burette reading [43]. For total phosphorus measurement, 70 µL of the 250 g L^−1^ Na_2_S_2_O_8_ solution was added to a brown glass bottle containing 25 mL of the soil sample and mixed. Then, these bottles were placed in a water boiler at 95 °C for 3 h. After digestion, 0.5 mL of the AA and MR solutions were added sequentially to the cooled digested solution, and mixed thoroughly. The characteristic blue color fully developed within 5 min at room temperature. The absorbance of the formed PMB compound was measured at 700 nm with a Thermo Scientific™ SPECTRONIC™ 200 Spectrophotometer (Thermo Fisher Scientific Inc., Shanghai, China) using a 5 cm cuvette [44]. For total potassium content measurement, add 25 mL ammonium acetate extracting solution to 5 g soil and shake it for 5 min. Filter the contents and collect the filtrate. Atomize the above extract on a flame photometer and record the readings [43].

### 4.6. Statistical Analysis

The means and standard errors with 3 replicates of the above experiments were analyzed by *t*-test or one-way variance analysis with IBM-SPSS 21.0 (IBM, Armonk, NY, USA), and S-N-K’s test was used to test the homogeneity of variance. The graph was drawn by OriginPro 2021 (OriginLab Corporation, Northampton, MA, USA).

## 5. Conclusions

It is safely indicated that the use of different rootstocks and scions had a significant influence on the survival rate of the grafted macadamia plants in China. HAES788 used as rootstock and Guire No. 1 used as scion are the most interesting findings in this study. The best grafting time was December. Among the 16 substrate formulations, substrates T12, T13, T15, and T16 were the best for grafted plant growth because they had the advantage of promoting a root soil structure more suitable for grafted plant development. These results provide a fundamental guide for a grafting program in macadamia cultivation.

## Figures and Tables

**Table 1:** Experimental design of substrate compositions (volume ratio).

	Proportion	Peat Soil	Perlite	Wood Bran	Loess
Substrates					
CK		0	0	0	10
T1		6	1	1	2
T2		6	1	2	1
T3		6	2	1	1
T4		5	1	1	3
T5		5	1	3	1
T6		5	3	1	1
T7		5	1	2	2
T8		5	2	1	2
T9		5	2	2	1
T10		4	2	2	2
T11		4	1	2	3
T12		4	2	1	3
T13		4	2	3	1
T14		4	3	2	1
T15		4	3	1	2
T16		4	1	3	2

## Data Availability

All data generated or analyzed are included in the article.
